# Risk factors for malnutrition among preschool children in rural Karnataka: a case-control study

**DOI:** 10.1186/s12889-018-5124-3

**Published:** 2018-02-26

**Authors:** Ansuya, Baby S. Nayak, B. Unnikrishnan, Anice George, Shashidhara Y. N., Suneel C. Mundkur, Vasudev Guddattu

**Affiliations:** 1 Department of Community Health Nursing, Manipal College of Nursing, Manipal Academy of Higher Education, Manipal, 576104 India; 2 Department of Pediatric Nursing, Manipal College of Nursing, Manipal Academy of Higher Education, Manipal, 576104 India; 3 Department of community medicine, Kasturba Medical College Mangaluru, Manipal Academy of Higher Education, Manipal, 576104 India; 4 Department of Pediatric Nursing, Manipal College of Nursing, Manipal Academy of Higher Education, Manipal, 576104 India; 5 Community Health Nursing, Manipal College of Nursing Manipal, Manipal Academy of Higher Education, Manipal, 576104 India; 6 Department of Pediatrics, Kasturba Hospital, Manipal Academy of Higher Education, Manipal, 576104 India; 7 Department of Statistics, Manipal Academy of Higher Education, Manipal, 576104 India

**Keywords:** Preschool children, Risk factors, Rural, Malnutrition, Under nutrition, Karnataka

## Abstract

**Background:**

The prevalence of malnutrition among children in developing countries is very high. As a step towards reducing the prevalence of malnutrition, there is a need to identify the important determinants of malnutrition in the specific population so that preventive and control measures can be implemented.

The objective of the study is to determine the risk factors for malnutrition among preschool children in Rural Karnataka, South India.

**Methods:**

A case-control study was carried out among preschool children, aged between three to six years, attending the Anganwadi centers and their mothers’ in Udupi district of Karnataka, India. A total of 570 children (190 cases and 380 controls) were selected by multistage cluster sampling technique. A semi-structured risk factors questionnaire was used to identify the risk factors for malnutrition among children.

**Results:**

The majority (45.8 and 45.5%) of the children in the study were in the age group of 3.0 to 4.0 years in case and control groups respectively. There was a slight preponderance of illiterate parents among cases in comparison to the controls. Largely, 87.4% of the children belonged to poor socio-economic status in the case groups compared to 82.4% in the control group. After adjusting for the confounders, underweight was significantly associated with socio-economic status of the parents (aOR: 2.05, 95% CI: 1.06, 3.96), birth weight < 2000 g (aOR: 25, 95% CI: 0.10, 0.59), recurrent diarrhoea (aOR: 2.74, 95% CI: 1.56, 4.83), recurrent cold and cough (aOR: 3.88, 95% CI: 1.96, 7.67), worm infestation (aOR: 2.0, 95% CI: 1.19, 3.38) and prelacteal feed given (aOR: 3.64, 95% CI: 2.27, 5.86).

**Conclusion:**

Parental education, childhood illness, short birth interval, open defecation, type of weaning and complimentary food given to children were some of the significant determinants of underweight that were found in the study. Information, Education and Communication (IEC) campaigns alleviating food habits and taboos and promoting birth spacing is the need of the hour for preventing the occurrence of undernutrition among preschool children.

**Electronic supplementary material:**

The online version of this article (10.1186/s12889-018-5124-3) contains supplementary material, which is available to authorized users.

## Background

Undernutrition is one of the utmost significant universal health problems, and it affects a large number of children in the developing countries [1]. Proper nutrition of children, leading to adequate growth and good health is the essential foundation of human development [2]. UNICEF, in the year 2006, reported the causes of childhood malnutrition as insufficient diet, frequent infections, poor breastfeeding practices, delayed introduction of complementary foods and inadequate protein in the diet. Other factors that influence food intake include health status, food taboos, growth and personal choice related to diet. Malnutrition can also develop due to neglect, abnormal mealtimes, insufficient quantities of food and insufficient parental knowledge [3].

Chronic under nutrition is associated with serious health impairments later in life. Under nutrition in young child results in delayed physical growth and motor development, impedes behavioral and cognitive development that results in diminished academic performance and social skills. Moreover malnutrition during early childhood leads to serious long term consequences later in life which increases risk of developing diseases or disabilities and even death. Despite of these consequences, malnutrition is a treatable with prompt identification, anticipation and management [3].

Malnutrition is largely a treatable condition. Therefore, prompt identification, prevention and treatment is vital. Malnutrition in children relics a substantial problem in India, in spite of global efforts on maternal child health improvement, and specific programmes such as Integrated Child Development Services (ICDS). The percentage of underweight, stunting and wasting among children under three years of age are reported to be 47%, 45% and 16% respectively in India [4]. There is also a wide disparity in the prevalence of undernutrition of children among the states of India, ranging from high (Madhya Pradesh - 55%) to relatively low (Tamil Nadu - 25%) [5]. National Family Health Survey-4 (2015–16) reported that the prevalence of underweight, stunting, wasting and severe wasting among children under five years is 31.5%, 32.6%, 24.8% and 9.7% respectively in Karnataka State [6]. District level household and facility Survey-4 (2015–16) reported that the prevalence of underweight, stunting, wasting and severely wasting among under-five children is 22.3%, 21.1%, 20.9% and 4.0% respectively in Udupi district [7]. Although Udupi district - an important town in coastal Karnataka, South India, has good health indicators, malnutrition among children is still a persisting problem [8].

Studies from Udupi district has reported the magnitude and the predictors of malnutrition. Kumar et al. (2010) conducted a cross-sectional study in Udupi taluk. The result showed that 32.3% children were malnourished, of which girls were 46.2% and boys 33.6% [9]. Chakravarthy, Soans and Hanumanth (2015) reported that underweight among children of 1–3 years was 14.8% [10]. Prabhat and Malya (2015) reported that the prevalence of underweight among 2–5 year old children was 46% [11]. A case-control conducted by Basit et al. (2012) reported that less birth spacing with more than two children in the family, low birth weight and sickness in the past one month to be significant predictors of under nutrition. There was significant association with under nutrition for a diet without milk or with diluted milk [12]. Many studies were conducted on fragmental basis, to find an association of nutritional status with either feeding practices or socio-economic status. Studies on other risk factors were not commonly attempted. Thus, this study was aimed to determine and analyze the comprehensive risk factors leading to under nutrition among preschool children attending Anganwadi centers.

## Methods

### Study design

A community-based case-control study was conducted in Udupi district of Karnataka, India.

### Study participants

The study was conducted among the dyad sample of preschool children between three to six years, attending Anganwadi centers and their mothers.

### Sampling technique and sample size

A Survey was carried out to identify the preschool children with malnutrition. Multistage cluster sampling technique was adopted to select the Anganwadi centers. Udupi taluk was selected from Udupi district of Karnataka state for the study. Fifteen villages (gramas) were randomly selected by chits from Udupi taluk. A cluster of 93 Anganawadi centers were selected from these 15 villages. A total of 1485 preschool children were assessed for nutritional status from 93 selected Anganwadi centres.

Among these 1485 children, 362 were identified as malnourished based on new WHO child growth standards 2006 (weight for age) [13].

Assuming 20% poor practices among the cases, anticipated odds ratio of 2%, at the power of 80% and considering 10% non-response, the sample size required for cases was 190. The control group was selected based on 1:2 ratio. Thus, a sample of 190 cases and 380 controls were included in the study. For each malnourished child identified (case), two normal weight children (controls), who met the inclusion criteria were selected immediate succeeding to child’s register number in the attendance register maintained in the Anganwadi centre.

### Measurements

Measurement of a weight of the preschool children was done to identify the malnutrition. The nutritional status was graded as per the new WHO child growth standards 2006 [13]. Children in the age group of 3–6 years, with weight for age ratio less than – 2 SD (− 2Z Scores) and not suffering from any chronic /severe illness were considered a case. Controls were healthy children in the same age group with weight for age ratio above − 2 SD (> − 2Z scores).

Data were collected using tools such as Demographic proforma, Socio-economic status scale and semi-structured Risk factors questionnaire [14, 15]. Demographic proforma and semi structured risk factors questionnaire was developed by the researcher initially in English and it was validated (CVI-1) and checked for the reliability (*r* = 0.96). Socio-economic status (SES) was assessed using a modified “Scale for measuring socio-economic status of a family” developed by O. P. Aggarwal & et al. [16]. Then tools were translated to Kannada language (local language), then re-translated to English to check the language validity (Additional file 1).

### Ethical approval

Ethical approval was taken from Institutional Ethics Committee (IEC 432/2013) to conduct the study. The permission was obtained from Child development project officer (CDPO) of Udupi taluk to visit Anganawadi centres and approach preschool children and their mothers. Written informed consent was obtained from the mothers for participation their children as well as their own participation.

### Procedure for data collection

After obtaining informed consent from the mothers, anthropometric measurements of the children were assessed using standard calibrated instruments. Weight was recorded using a standard calibrated weighing scale, kept on a firm horizontal surface to the nearest 500 g with zero error. Based on the recorded weight, nutritional status was graded as per the new WHO child growth standards 2006 [13]. Mothers of selected children were contacted at their residence to collect the information on risk factors. Initial rapport was developed with the mothers. They were asked to fill up the questionnaire. Information pertaining to risk factors such as childbirth history, illness history, environmental factors, and feeding practices of the child were obtained using a semi-structured risk factors questionnaire. The average time taken by each mother to respond to the questionnaires is 25 min.

### Data analysis

The collected data were analyzed using Statistical Package for Social Sciences (SPSS) version 16. The findings were reported in terms of frequency and percentage, along with 95% confidence interval (CI). The risk was estimated using odds ratio with 95% CI. Univariate and multivariate logistic regression was done to identify the risk factors for under nutrition.

## Results

The socio-demographic characteristics of the study population are presented in Table 1.

**Table 1:** Demographic characteristics of the participants (*N* = 570)

Demographic characteristics	Cases *n* = 190 (%)	Controls *n* = 380 (%)
Age in years		
3.0–4.0	87 (45.8)	173 (45.5)
4.1–5.0	63 (33.2)	138 (36.3)
5.1–6.0	40 (21.1)	69 (18.2)
Gender		
Male	85 (44.7)	184 (48.4)
Female	105 (55.3)	196 (51.6)
Religion		
Hindu	182 (95.8)	351 (92.4)
Christian	5 (2.6)	6 (1.6)
Muslim	3 (1.6)	23 (6.1)
Type of family		
Nuclear	110 (57.9)	214 (56.3)
Joint/Extended	80 (42.1)	166 (43.7)
Education of father		
Illiterate	18 (9.5)	5 (1.3)
Primary and <10th std.	146 (76.8)	301 (79.2)
10th std. and below graduation	25 (13.2)	72 (18.9)
Graduation and above	1 (0.5)	2 (0.5)
Education of mother		
Illiterate	13 (6.8)	6 (1.6)
Primary and <10th std.	143 (75.3)	269 (70.8)
10th std. and below graduation	34 (17.9)	104 (27.4)
Graduation and above	0	1 (0.3)
Caretaker		
Mother	187 (98.4)	372 (97.9)
Grandmother/Other member in the family	3 (1.6)	8 (2.1)
Socio-economic status		
Middle	24 (12.6)	67 (17.6)
Poor/BPL	166 (87.4)	313 (82.4)

The data presented in Table 1 shows that, there was an almost equal distribution among the cases and controls, with regard to characteristics such as age, gender, religion and family type. It was observed that, majority of the children, i.e., 45.8% among cases and 45.5% among controls were in the age group of 3 to 4 years. There was a slight preponderance of illiterate parents among cases. In both the groups, large number (87.4% in cases and 82.4% in control) of children were belonged to poor socio-economic status, whereas none of the children were from higher socio-economic status.

### Univariate analysis of risk factors for malnutrition

Chi-square was computed to find the association between malnutrition and risk factors. A logistic regression of 95% confidence interval was then carried out to adjust for the confounders and identify the factors that were truly associated with malnutrition.

Socio-demographic determinants: Gender, type of family, immunization status of the child, education and socio-economic status of the parents were the factors focused on this area. Statistically, a significant association was found between malnutrition and immunization status of the child as well as the educational status of mother (χ^2^_(df)_ = 15.8_(3_), *p* < 0.001) and the father (χ2_(df)_ = 22.2_(3)_, *p* < 0.001). A child’s risk of malnutrition was higher when he/she was partially immunized, as compared to a child who was completely immunized [OR 2.31, 95% CI (1.58–3.36) *p* < 0.001].

The Child related risk factors presented in Table 2 depicts that children with birth weight less than 2000 g were 1.9 times, and those between 2000 and 2500 g were 3.9 times at a higher risk of being malnourished, as compared to children with birth weight more than 2500 g. Second and third birth order children were 3.8 times and 2.7 times higher risk of being malnourished as compared to the first born. The birth interval between the first and the second child and between the second and the third child, if less than 3 years, had a high risk of malnutrition (p < 0.001).

**Table 2:** Child related risk factors for malnutrition

Factors	Case *n* = 190 (%)	Control *n* = 380 (%)	Odds ratio (95%CI)	*p* value
Child born				
Preterm	36 (18.9)	65 (17.1)		0.587
Full term	154 (81.1)	315 (82.9)		
Birth weight in grams				
<2000	24 (12.6)	16 (4.2)	1.97 (0.95–4.07)	
2000-2500	54 (28.4)	71 (18.7)	3.92 (2.01–7.66)	0.001
>2500	112 (58.9)	293 (77.1)	1	
Birth order of the child				
First	112 (58.9)	253 (66.6)	1	
Second	66 (34.7)	119 (31.3)	3.38 (1.34–8.51)	0.018
Third and more	12 (6.3)	8 (2.1)	2.70 (1.05–6.95)	
Birth interval (from previous birth)				
First born	54 (28.4)	167 (43.9)		
less than or 1 year	5 (2.6)	3 (0.8)	0.28 (.17–.48)	
1 to 2 years	45 (23.7)	40 (10.5)	0.50 (.31–.80)	0.001
2 to 3 years	50 (26.3)	78 (20.5)	0.82 (.50–1.35)	
> 3 years	36 (18.9)	92 (24.2)	1	
Birth interval between 2nd and 3rd child				
First born and 2nd child	163 (85.8)	361 (95.0)		
2 and less years	17 (8.9)	5 (1.3)	0.35 (.07–1.66)	0.001
2–3 years	4 (2.1)	9 (2.4)	2.70 (0.50–14.3)	
> 3 years	6 (3.2)	5 (1.3)	1	

The Child illness factors are shown in Table 3. A malnourished child was noted to have 6.9 times higher risk of having suffered from recurrent cold and cough, and 10 times the risk of having recurrent diarrhea in the previous year. Also, a malnourished child has 4.6 times and 6.8 times higher risk of having suffered from worm infestation and poor appetite respectively.

**Table 3:** Association between Child illness and Malnutrition

Factors	Case *n* = 190(%)	Control *n* = 380 (%)	Odds ratio (95%CI)	*p* value
History of present Illness of the child				
No	179 (94.2)	367 (96.6)		0.184
Yes	11 (5.8)	13 (3.4)	1.73 (0.76–3.94)	
Had Chronic infection (past)				
No	183 (96.3)	371 (97.6)		0.370
Yes	7 (3.7)	9 (2.4)	1.57 (0.57–4.30)	
Recurrent diarrhoea				
No	110 (57.9)	344 (90.5)		0.001
Yes	80 (42.1)	36 (9.5)	6.94 (4.44–10.87)	
Recurrent cold and cough				
No	12 (6.3)	152 (40.0)		0.001
Yes	178 (93.7)	228 (60.0)	9.99 (5.38–18.57)	
Poor appetite				
No	67 (35.3)	300 (78.9)		0.001
Yes	123 (64.7)	80 (21.1)	6.88 (4.67–10.13)	
Had worm infestation in the past				
No	107 (56.3)	326 (85.8)		0.001
Yes	83 (43.7)	54 (14.2)	4.68 (3.11–7.03)	

### Environmental risk factors (water and sanitation characteristics)

Children practicing open defecation were more among cases (14.7%) than in controls (6.8%), and they also tend to have a 2.3 times higher risk of being malnourished, compared to children using a sanitary latrine [95%CI (1.37–4.14), *p* = 0.002]. Children whose families had open drainage system around the house was noted to be more in cases (74.7%) than controls (58.2%), and were at 2.0 times at a higher risk of being malnourished than families that had underground and piped drainage system [95%CI (1.329–3.29), *p* < 0.001. Factors such as a source of water and method of water storage in the house did not have any association with malnutrition, but the method of extracting the drinking water (for example, immersing both the glass and the hand into the stored water) by the children had the significant association with malnutrition. Children who had the habit of immersing both their hand and the glass to extract drinking water, and children who had the habit of immersing only the glass and not their hand, was noted to be more in cases compared to controls. They had 4.7 times [95%CI 2.02–11.05, *p* < 0.001] and 7.25 times [95%CI: 3.00–17.49, *p* < 0.001] higher risk, respectively, of being malnourished as compared to children using a long spoon to extract drinking water from a stored vessel.

Table 4 depicts the association of infant feeding and dietary practices with malnutrition, which shows that among cases, 60% of the mothers gave a prelacteal feed (either plain water, sugar water or glucose water) to their children, as compared to 20.3% in controls. This was found to have a 5.9 times higher risk of being malnourished [95%CI 4.02–8.6, *p* < 0.001]. The proportion of children who were exclusively breastfed up to six months were higher among cases (58.9%), and those children who received exclusive breastfeeding more than six months were higher among controls (56.8%). and the risk of being malnourished was 1.89 [95%CI 1.32–2.69, *p* < 0.001] times. Neither the time of initiation of weaning nor the time at which the complementary food was given (*p* = 0.065) influenced the nutritional status, but the type of weaning (*p* < 0.001) and the type of complementary food given (*p* = 0.003) highly influenced the nutritional status of the children. The mothers who restricted certain foods for their children during childhood were found to have a statistically significant association (*p* < 0.001) of having malnourished children with an Odds ratio of 2.57. Likewise, mothers who had the practice of providing nutritious food to their children during their infancy and childhood, were found to have normal weight children, as compared to the mothers who had the practice of giving their children bakery products and candies or chocolates (*p* < 0.001).

**Table 4:** Association of Infant feeding and dietary practices with malnutrition

Factors	Case *n* = 190 (%)	Control *n* = 380 (%)	Odds ratio (95%CI)	*p* value
Colostrum given				
Yes	177 (93.16)	365 (96.1)		0.111
No	13 (6.84)	16 (3.9)		
Pre-lacteal feed				
No	76 (40.0)	303 (79.7)		0.001
Yes	114 (60.0)	77 (20.3)	5.90 (4.02–8.6)	
Exclusive breast feed given				
Up to 6 months	112 (58.9)	164 (43.2)	1.89 (1.32–2.69)	0.001
6 and more than 6 months	78 (41.1)	216 (56.8)		
Type of weaning food				
Rice	48 (25.3)	59 (15.5)	1.41 (0.89–2.22)	0.001
Rice, milk	100 (52.6)	171 (45.0)	2.81 **(**1.69–4.6**)**	
Rice, milk, veg/pulses	42 (22.1)	150 (39.5)		
Type of complimentary food given				
Veg/pulses	16 (8.4)	14 (3.7)	0.30 (.14–.66)	0.003
Veg/pulses and egg/fish	119 (62.6)	209 (55.0)	0.61 (.42–.90)	
Veg/pulses, egg/fish and meat/ chicken	55 (28.9)	157 (41.3)		
Bottle feed				
No	137 (72.1)	281 (73.9)		0.639
Yes	53 (27.9)	99 (26.1)		
Food restriction				
No	109 (57.4)	295 (77.6)		0.001
Yes	81 (42.6)	85 (22.4)	2.57 (1.7–3.75)	
Special food prepared and given				
No	161 (84.7)	271 (71.3)		0.001
Yes	29 (15.3)	109 (28.7)	2.23 **(**1.41–3.51)	
Frequency of candies/chocolate given				
Daily/alternative days	143 (75.3)	228 (60.0)	2.02 **(**1.37–2.99)	0.001
Once/twice week	47 (24.7)	152 (40.0)		

Further, to identify the independent risk factors, multiple logistic regression with a stepwise Backward LR was computed and expressed as adjusted odds ratio at 95% confidence interval, are presented in Table 5.

**Table 5:** Adjusted odds ratio of risk factors for malnutrition - a multivariable analysis (*N* = 570)

Variables at risk	Adjusted Odds ratio (95%CI)	*p* value
Socio-economic status	2.05 (1.06–3.95)	0.031
Birth weight < 2000 g	0.25 (0.10–0.59)	0.002
Birth weight 2000 - 2500 g	0.52 (0.30–0.91)	0.022
Recurrent diarrhoea	2.74 (1.56–4.82)	0.001
Recurrent cold and cough	3.88 (1.96–7.67)	0.001
Less of appetite	4.90 (3.03–7.93)	0.001
Worm infestation	2.00 (1.19–3.38)	0.009
Prelacteal feed	3.64 (2.27–5.86)	0.001
Special food prepared and given	0.50 (0.28–0.88)	0.016

A logistic regression analysis confirmed the factors such as socioeconomic status of the parents, birth weight < 2500 g, recurrent diarrhea, recurrent cold and cough, poor appetite of the child, worm infestation, prelacteal feed and special food prepared as the risk factor for Malnutrition.

## Discussion

Malnutrition is a multi-dimensional entity. The nutritional status of children under the age of five is affected by different factors. The present study identifies certain risk factors which were found to be significantly higher in children with malnutrition compared to normal children. In our study we found that the educational status of the parents, was associated with the nutritional status of the child, these findings are consistent with earlier reports [17–21].

The socio-economic status of the family was independently associated with under-nutrition as the study population were from rural areas, a supportive study done in India and Africa reveals that families with low economic status have significant association with under nutrition [22, 23].

As per the recommendation of global public health, for achieving optimum growth, development and health a child should be breast fed exclusively during the first six months of life. To evolve as a healthy individual, the infant should be continued with adequate and appropriate safe complimentary food along with breast milk up to two years of age or beyond [24]. In the present study, it was found that exclusive breastfeeding for less than six months was higher among cases (58.9%) than controls (43.2%), and was associated with the nutritional status of the children. It was also noted that the chances of being malnourished were 1.89 times higher among those children who did not receive exclusive breastfeeding. Various studies have shown that lack of exclusive breastfeeding for the first six months was significantly associated with malnutrition [18, 19, 25, 26]. A study done in Bangladesh reported that there was a four-fold increased risk of malnutrition with the lack of breastfeeding [27].

WHO (2000) has reported that the children who are underweight are at an increased risk of mortality from infectious illnesses such as diarrhea and pneumonia. Infections play a major etiological role in under nutrition because they result in increased needs and high energy expenditure, lower appetite, nutrient loss due to vomiting, diarrhea, poor digestion, malabsorption and the utilization of nutrients and disruption of metabolic equilibrium [28, 29]. In this study, no association was found between children suffering from current or chronic infections (such as tuberculosis, malaria, etc.) in the past and the nutritional status. But the risk of malnutrition was independently associated with recurrent cold and cough, recurrent diarrhoeal illness, poor appetite and worm infestation that occurred over the previous year. Multivariate logistic regression analysis reiterated the association of these factors with malnutrition. The study showed the high prevalence of current infection among those who were malnourished, and it was suggested that the malnourished children had a higher incidence of infections due to poor immune factors as a result of inadequate nutrition. This is consistent with the findings of a study done in Karnataka, India [12]. The relationship between the child’s nutritional status and illness is bi-directional [30]. Being underweight increases the likelihood of illness because malnutrition suppresses immunity [31]. Conversely, an acute infection can lead to weight loss through the increase in metabolic demand, impaired nutrient absorption or anorexia [32, 33].

The present study showed the association of low birth weight i.e., < 2500 g, birth order of second or third and a birth interval of fewer than three years with malnutrition, which is consistent with other studies. Studies were done in India and Bangladesh also reported that low birth weight and inadequate birth spacing as a risk factor for malnutrition [12, 27].

The current study revealed that environmental factor is also a strong predictor of malnutrition. A higher number of children who practiced open defecation were found in malnourished children (14.7%) than the normal (6.8%). Unhygienic conditions such as an open drainage around or near the house and waste dumped near the house, practices such as the children and the household members drinking unprotected water or drinking the water stored in open containers, immersing both the glass and the hand while extracting drinking water, etc., was observed more in a malnourished child. Studies also reported that poor sanitation status, lack of personal hygiene and low socioeconomic statuses have an impact on malnutrition. Unhygienic latrines, defecation within premises and use of unprotected surface water have an increased association with malnutrition. Children from unclean households were more underweight (*p* = .037) than the children whose households were clean [29, 34, 35]. It revealed that poor environmental conditions may increase the risk of acquiring an infectious disease, which in turn may lead to malnutrition.

Malnourishment is also associated with pre-lacteal feeding. In the present study the risk of developing malnutrition is 3.64 times higher (*p* = 0.001) among the children who were given pre-lacteal feeds. Many studies conducted in India also reported higher percentage of infants were given pre-lacteal feeds [36, 37].

There is an increased risk of malnutrition, either with an early introduction or with delayed initiation of complementary feeding. Independent association of malnutrition with the consistency of complementary feeds was found in studies from other parts of India [18, 25]. Our study showed that the type of weaning and the complementary feeding highly influenced the nutritional status of the children. But, the study failed to show any association between the time of introduction of weaning and the time complementary feeding with the nutritional status of the children. Similar findings were reported in another study [18]. The study also found an association between food restrictions and special food fed and malnutrition among the children.

A higher percentage of sweets and candies/chocolate consumption among the children was observed among malnourished children as compared to normal children. The higher risk of malnutrition among children who ate more sweets or candies might be due to the lack of intake of nutritious food which is required for growth of the child.

## Conclusion

As the quality of the future human resources depends on the children of today, improvement of their nutritional level should be given top priority. Being underweight is associated with many factors which at times modifiable and at times non-modifiable. Considering the modifiable factors, the nutritional level can be improved. There should be some training or education about nutritional knowledge, environmental sanitation and personal hygiene, breastfeeding and weaning practices, nutritional deficiency diseases, the nutritional value of food and dietary practices to increase the awareness of rural parents to feed their children with a balanced diet, so that they can easily overcome the problems of malnutrition.

## Additional file

Additional file 1: Questionnaire. (PDF 269 kb)

## Abbreviations

aOR: Adjusted Odds Ratio
CDPO: Child development project officer
CI: Confidence Interval
ICDS: Integrated Child Development Services
IEC: Information, Education, and Communication
OR: Odds Ratio
SD: Standard Deviation
SES: Socio-Economic Status
SPSS: Statistical Package for Social Sciences
UNICEF: United Nations Children’s’ Fund
WHO: World Health Organization
