# Supplementary material for: Risk factors for malnutrition among preschool children in rural Karnataka: a case-control study
Source: BMC Public Health. 2018 Feb 26;18:283. doi: 10.1186/s12889-018-5124-3 (PMC5828120; doi:10.1186/s12889-018-5124-3)
Supplement: Supplementary file 1 — Questionnaire. (PDF 269 kb) [file 12889_2018_5124_MOESM1_ESM.pdf]

Tool 1 :Background Information

Instruction : Please put tick mark (√) orfill the below given questions in the space provided.

Informant :

Section A: Demographic Proforma: Code No:

1. Date of birth :
2. Age of the child ----- years ----- months
3. Gender : Male/Female
4. Religion: Hindu/ Muslim / Christian/others(specify)…….
5. Type of family: Nuclear / Joint/Extended
6. Child is taken care by : mother/grandmother/ other members in the family.

Section B : Anthropometric measurement

Weight : -------------------Kgs

Height :--------------------cm

Section C :Immunization Status

Vaccine	At birth	1 ½ Months	2 ½ months	3 ½ months	9months	16-24months	5-6 years
OPV							
BCG							
DPT							
Measles							
Vitamin A							
Hepatitis B							

Tool 2: Questionnaire on Risk factors for Malnutrition

Note: This tool has six parts.

Part I :Scale for measuring socio-economic status of Family

(Modified from O.P. Aggarwal & et. al)

Instruction : Various alternatives for each question are given in this questionnaire. You are requested to select one alternative and put a tick (√) mark in the space provided.

1. What is the monthly per capita income from all sources (total monthly income /no. of family members)

1. >50000	( )
2. 20000-49999	( )
3. 10000-19999	( )
4. 5000-9999	( )
5. 2500-4999	( )
6. 1000-2499	( )
7. <1000	( )

2. What is the educational status of child's father?

1. Professional qualification	( )
2. Post-graduation	( )
3. Graduation	( )
4. 10th class pass but less than Graduation	( )
5. Primary pass but less than 10th	( )
6. Less than Primary but attended school for at least one year	( )
7. Just literate but no schooling	( )
8. Illiterate	( )

3. What is the educational status of child's mother?

1. Professional qualification	( )
2. Post-graduation	( )
3. Graduation	( )
4. 10th class pass but less than Graduation	( )
5. Primary pass but less than 10th	( )
6. Less than Primary but attended school for at least one year	( )
7. Just literate but no schooling	( )
8. Illiterate	( )

4. What is the occupation of child's father?

1. Service in central/State/Public undertakings or Owner of a company employing>20 persons or self-employed professional viz Doctors, CAs, Eng. Etc.	( )
2. Service in Private sector or independent business employing 2-20 persons	( )
3. Service at shops, home, transport, own cultivation of land	( )
4. Self employed e.g. shops, Rehdies or petty businesswith income >5000	( )
5. Self-employed with income <5000(labourer, house wife)	( )

5. What is the occupation of child's mother?

1. Service in central/State/Public undertakings or Owner of a company employing>20 persons or self-employed professional viz Doctors, CAs, Eng. Etc.	( )
2. Service in Private sector or independent business employing 2-20 persons	( )

3. Service at shops, home, transport, own cultivation of land ( )
4. Self-employed>5000 ( )
5. Self-employed<5000(labourer, house wife) ( )

6. Family possessions (presence of each item given below will carry score of .1.)
1. Refrigerator
2. TV
3. Radio/Transistor/Music system
4. AC
5. Washing Machine
6. Telephone
7. Mobile, telephone
8. Credit card
9. Sanitary latrine.
10. Any newspaper subscribed throughout the month

7. Type of living house
1. Own house with 5 or more rooms ( )
2. Own house with 3-4 rooms ( )
3. Rented/Govt. house with 5 or more rooms ( )
4. Own house with 1-2 rooms ( )
5. Rented/Govt. house with 3-4 rooms ( )
6. Rented/Govt. house with 1-2 rooms ( )
7. No place to live, pavement, mobile cart ( )

8. Possession of a vehicle or equivalent
1. 2 or more cars/Tractors/Trucks ( )
2. 1 Car /Tractor/Truck ( )
3. 1 or more scooter(s)/Bullock cart (s) ( )
4. 1 or more cycles (not baby cycle) ( )
5. None of the above ( )

9. No. of earning members in the family (Nuclear/Joint)
1. 3 or more members earning ( )
2. 2 or both husband and wife earning ( )
3. Only 1 family member earning ( )
4. No earning member ( )

10. No. of children, the head of the family has/had
1. 0-1 ( )
2. 2 ( )
3. 3 ( )
4. 4 ( )
5. 5 ( )
6. >6 ( )

11. Facility of some essentials in the family
1. Tap water supply ( )
2. Electricity ( )
3. None is present ( )

12. Employment of a domestic servant at home

1. Employed >2 full time servants on salary for domestic work ( )
2. Employed only 1 full time servant on salary for domestic work ( )
3. Employed > 3 part time servants on salary for domestic work ( )
4. Employed 1-2 part time servants on salary for domestic work ( )
5. Employed no servants for domestic work ( )

13.Type of locality the family is residing

1. Living in urban locality ( )
2. Living in rural locality ( )
3. Living in resettlement colony ( )
4. Living in slums ( )
5. No fixed living and mobile ( )

14. Members of family gone abroad in last three years(official or personal)

1. Whole family ( )
2. Only husband and wife ( )
3. Only 1 family member ( )
4. None ( )

15. Possession of agricultural land for cultivation

1. Own agricultural land >100 acres ( )
2. Own agricultural land 51-100 acres ( )
3. Own agricultural land 21-50 acres ( )
4. Own agricultural land 6-20 acres ( )
5. Own agricultural land 1-5 acres ( )
6. No agricultural land ( )

16.Presence of milk cattles in the family for business or non-business purposes

1. Own 4 or more milkcattles ( )
2. Own 1-3 milk cattles ( )
3. Own 1 milk cattle ( )
4. Does not own any milk cattle ( )

17.Positions held by any one member in the family in any organization

1. Holding position of 3 or more official or non-official organizations. ( )
2. Holding position of 1-2 official or not-official organizations ( )
3. Only participate in the activities of official or non-official organization ( )
4. Does not hold any such position ( )
5. Does not participate in any such activities ( )

Part II : Factors related to Child birth:

Instruction : You are requested to select one alternative and put tick ((√)) mark /fill the below given questions in the space provided. Please be free enough to give frank response.

1.Child born as : Preterm/ Full term/Post term

2.Birth weight : ------ grms / Do not know

3. No. of members in the family : -------------------

4.Birth order of this child : First / Second / Third/ Fourth.

(If birth order is not first, answer question no.6)

5. No. of siblings :-----------------------

6. Spacing between children:

Birth order of the child	Age	Spacing
1st child		
2nd child		
3rd child		
4th child		

Part III :Child Illness history :

1. Is the child suffering from any illness at present? : Yes/No

If yes, specify _________

2 Is the child suffering from any chronic infection ? : Yes/No

If yes, specify__________

3 Does the child have recurrent diarrhea (more than 4times passing loose stool/day)? Yes/No

If yes when was the last episode? ---------

How many episodes in a year?--------

4 How often does the child get diarrhea?---------------------

5 Does child get recurrent cough and cold (cough and cold for two weeks)?Yes/No

If yes, how often child gets? --------

How many episodes in a year?--------

6. Did the child ever get malaria in the past? Yes/No

If yes, How many times infected with malaria?-------------

7. Did the child ever get Tuberculosis in the past? Yes/No

If yes, specify the details---------------

8. Does your child has loss of appetite? Yes/No

9. Level of physical activity of the child Poor/Moderate/Good(Normal)

Part IV: Worm infestation

1. Does the child pass worms in stool? Yes/No

2. Did your child have worm infestation in the past? Yes/No

If yes, is the stool examination done? Yes/No

If yes, when was it done?

3. Did your child receive treatment for worm in stool? Yes/ No

If yes, when was the last dose given? ---------

How often is deworming done?-------

4. Findings of stool examination:

Occurrence : Positive ( )
Negative ( )

If positive : Type of worms : Round worm ( )
Pin worm ( )
Tape worm ( )
Any other -----------

Part V : Environmental sanitation :

1. Which method/ type of latrine does your family/child use?

a) Sanitary ( )
b) Open field ( )

2. The type of drainage system around the house is

a) Underground ( )
b) Through pipe ( )
c) Open to land ( )

3. The method practiced to dispose the waste is

a) Burning ( )
b) Putting in pit ( )
c) Dumping ( )

4. The material used for flooring is

a) Marble/Granite ( )
b) Cement ( )
c) Mud with cow dung ( )

5. The source of water supply to the house is

a) Tap water ( )
b) Hand pump ( )
c) Well with top covered ( )
d) Open well ( )
e) Lake ( )

6.Water used for drinking is

a) Boiled and cooled ( )
b) Directly from public tap ( )
c) Directly from well ( )
d) filtered/aqua guard ( )

7. Water is stored in

a) Closed container ( )
b) Open container ( )

8. How do you take water from the large container to the glass?

a) Using long handle spoon ( )
b) Directly from drinking glass ( )
c) Immersing hand and glass while taking water ( )

VI. Dietary practices :

Sl No.	Item			
1.	What type of diet do you consume? : Vegetarian/Non vegetarian/Mixed			
2.	**Neonatal feeding practice:**			
2.1	Was the child breast fed?	Yes	No	
2.2	Did you give first yellow thick milk (colostrum) to the child for first three days?	Yes	No	
2.3	Did you give any other feeds (pre-lacteal) before starting breast feeding?	Yes	No	
2.4	How did you breast feed your child? On demand/Once in 2 hours/Once in 4 hours			
2.5	How long was the child exclusively breast fed?---------year -------months			
3	**Infant Feeding practice:**			
3.1	When was weaning started for the child? --------------------			
3.1.1	What type of weaning food was given?<br>Cow'milk/ soft cooked rice/ fish/chicken/fruit juice/dhal/ vegetables			
3.2	Did you introduce other milk to your child? (cow milk, lactogen etc.)	Yes	No	
3.2.1	If yes, what type of milk did you give your child?<br>Cow's milk/Buffalo milk/ Tin milk/ dairy milk/ Any other specify-----------			
3.3	Was the child fed on bottle?	Yes	No	
3.3.1	If yes, How long /how many months did your child receive bottle feed?-------			
3.4	How do you clean the bottle?<br>Boiled in water after every feed/Boiled in water once a day/cleaning with cold water			
3.5	At what age complimentary feed started?-------------------------			
3.5.1	What type of complimentary food was given?			
3.6	When did you stop breast feeding?<br>At the age of child ------yrs------month			
3.7	When was regular diet started? ----------------------------------			
3.8	Did you restrict your child from accessing any type of food which you think is not good for the child?	Yes	No	

	(eg : ragi, fish, pulses etc….)		
3.8.1	If Yes, specify the food which you don't give and why?-------------------------------		
4	**Current practice :**		
4.1	How many meals does your child take per day: 3 meals / 2 meals / one meal		
4.2	How many times snacks are given at home per day : One time/ Two times / Three times / more than 3 times		
4.3	Do you prepare some special /Nutritious food to your child? Yes / No	Yes	No
4.3.1	If yes. Specify the nutritious food.---------- How often this is prepared?		
4.4	Do you wash hands and plates before feeding the child?	Yes	No
4.5	Do you force your child to eat nutritious food	Yes	No
4.6	Do you give bakery eatables to your child?	Yes	No
4.7	Do you offer chocolate/candy to your child? If yes, How frequently ? Daily / alternative days/ weekly twice/weekly once	Yes	No
4.9	Usually what drink do you prefer to give to your child? Milk/Fruit juice/Water/Tea/Coffee. If any other, Specify-----		
